# Forecast of the COVID-19 Epidemic Based on RF-BOA-LightGBM

**DOI:** 10.3390/healthcare9091172

**Published:** 2021-09-06

**Authors:** Zhe Li, Dehua Hu

**Affiliations:** School of Life Sciences, Central South University, Changsha 410083, China; zhanghang22@csu.edu.cn

**Keywords:** COVID-19, Baidu index, random forest, bayesian optimization, LightGBM

## Abstract

In this paper, we utilize the Internet big data tool, namely Baidu Index, to predict the development trend of the new coronavirus pneumonia epidemic to obtain further data. By selecting appropriate keywords, we can collect the data of COVID-19 cases in China between 1 January 2020 and 1 April 2020. After preprocessing the data set, the optimal sub-data set can be obtained by using random forest feature selection method. The optimization results of the seven hyperparameters of the LightGBM model by grid search, random search and Bayesian optimization algorithms are compared. The experimental results show that applying the data set obtained from the Baidu Index to the Bayesian-optimized LightGBM model can better predict the growth of the number of patients with new coronary pneumonias, and also help people to make accurate judgments to the development trend of the new coronary pneumonia.

## 1. Introduction

During the outbreak of infectious diseases, social media is usually the most active platform for the exchange of information on infectious disease, and the information released is often of good real-time. Using Internet information to predict the epidemic situation of infectious diseases is one of the current research hotspots. L. Lu et al. used Baidu index and micro-index to conduct a comparative study on influenza surveillance in China [1]. J. H. Lu, School of Public Health, Sun Yat-sen University, and others studied the use of Internet search queries or social media data to monitor the temporal and spatial trends of the Avian Influenza (H7N9) in China, and the results show that the number of H7N9 cases is positively correlated with Baidu Index and Weibo Index search results in space and time [2]. J. X. Feng of the University of South Georgia and others studied the impact of Chinese social networks on the Middle East Respiratory Syndrome Coronavirus and Avian Influenza [3]. Mutual relations prove the effectiveness of using social media to predict infectious diseases. H. G. Gu et al. collected data on cases of H7N9 avian influenza in the Chinese urban population through the Internet, as well as geographic and meteorological data during the same period, and established a disease risk early warning model for human infection with H7N9 avian influenza, which can identify the high risk areas of avian influenza outbreaks and issue an early warning [4]. However, in these studies, most of the search process of network data adopts manual empirical methods to select keywords for search, and the choice of keywords often has a greater impact on search results.

At present, the focus of the world’s attention is mainly on the changes in the epidemic situation of the new type of coronary pneumonia. During the four months after the outbreak of the new type of coronavirus in Wuhan, Hubei in December 2019, the epidemic information was widely disseminated on social media such as Baidu, Sina, 360, Sogou, WeChat and QQ. Google, Weibo, Zhihu, Dingxiangyuan, Twitter, Facebook, etc. also released a lot of information about the new coronavirus epidemic, especially through the Google platform to spread to the world. On 31 March 2019, Google launched a project called “COVID-19 Public Datasets” to provide a public database related to the epidemic and open it to the public for free, which means that people can freely access and analyze relevant data and information [5]. How to use this information to predict the spread of the new type of coronary pneumonia in time is an urgent research topic. Currently, X. M. Zhao and others have proposed to use big data retrospective technology to study the spreading trend and epidemic control of the new coronary pneumonia [6]. B. McCall et al. used artificial intelligence methods to predict the new type of coronary pneumonia, thereby protecting medical staff and controlling the spread of the epidemic [7]. These studies are still in the preliminary stage, and the use of network data and prediction of the new coronary pneumonia are not yet ideal.

In this article, we consider that the amount of data indexed by Baidu is large enough for us to use. Based on this, we use the first feature in the search index, namely Baidu index [8], to study the prediction of the epidemic of new coronary pneumonia. We collected data on COVID-19 cases in China from 1 January 2020 to 1 April 2020, and used the random forest feature selection method to select the optimal sub-data set, and used grid search, random search and the Bayesian optimization algorithm optimizes the 7 hyperparameters of the LightGBM (light gradient boosting machine) model. The results show that the application of the data set obtained from the Baidu index to the Bayesian-optimized LightGBM model can better predict the growth of the number of patients with new coronary pneumonia.

This paper is organized as follows. In Section 2, we introduce the data set and analysis method used in detail. Baidu index search and actual case results are compared in time and space, and the impact of keywords and selected index in Baidu index search on the results is analyzed. Model structure, data set preprocessing methods, tuning algorithm, etc. are also introduced in detail. In Section 3, the experimental results are showed and related discussions are presented. Finally, the conclusion is drawn in Section 4.

## 2. Materials and Methods

### 2.1. COVID-19 Dataset

In order to standardize prevention and treatment, on 11 February 2020, the World Health Organization named the pneumonia caused by the new coronavirus as “COVID-19” (Corona Virus Disease 2019). In this study, we first obtain the data of COVID-19 cases that occurred in China from 1 January 2020 to 1 April 2020 by searching the COVID-19 Public Datasets on the Google platform, mainly including diagnosis number and death toll, and use them as actual data. These data are released by the Centers for Disease Control (CDC), so we identify these data as CDC data , namely the CDC-Diagnosis and CDC-Death toll mentioned in this paper. Then, we can collect keywords related to COVID-19 through commonly used social networking sites, such as Baidu, Sina, 360, Sogou, WeChat, QQ, Google, Weibo, Zhihu, Dingxiangyuan, Twitter, Facebook, etc., And form a keyword library. Then use the Baidu index platform (http://index.baidu.com, (accessed on 1 April 2020)) to retrieve relevant keywords, and use the statistics of the average daily search volume of relevant Chinese keywords as social network mining data for prediction. In this article, this part of the data is identified as Baidu index data.

By searching for the name and clinical symptoms of new coronavirus pneumonia on social networking sites, we can get the following keywords: new coronavirus, fever, dry cough, fatigue, dyspnea and cough. Using the Baidu index platform to retrieve the above keywords, we can get the average daily search volume of each keyword from 1 January 2020 to 1 April 2020, that is, Baidu index data. Table 1 shows part of the data of the CDC data set and the Baidu index data set. See Appendix A for all the data.

#### 2.1.1. Time and Space Comparative Analysis of Baidu Index Search and Actual Cases

**Figure 1:**
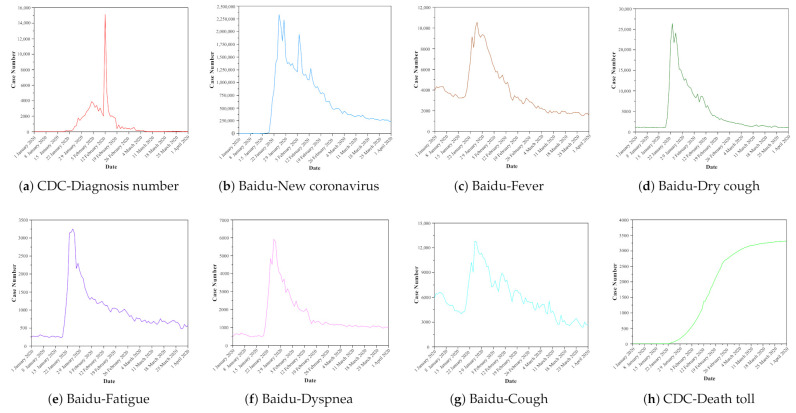
The number released by the CDC and Baidu index data based on keyword searches. (**a**) represents the number of newly diagnosis released by the CDC. (**b**–**g**) represent the Baidu index data based on keywords “New coronavirus”, “Fever”, “Dry cough”, “Fatigue”, “Dyspnea”, “Cough” respectively. (**h**) represents the death toll released by the CDC. CDC = Centers of Disease Control.

Based on the data obtained during the data collection phase, we have drawn the trend graph of CDC data and Baidu Index data over time, as shown in Figure 1. From Figure 1a–g, it can be seen that the keyword “dry cough” is the most commonly used keyword when Chinese netizens search for symptoms of new coronavirus pneumonia, followed by fever, dyspnea, and fatigue. We can see that in the Baidu index method, the keywords “new coronavirus” and “dry cough” are the best choices. The extracted data has the best spatio-temporal positive correlation with the actual number of cases. Through website search, we can find that these two keywords mainly appear in the columns of Baidu Baike and Baidu Health Pharmacopoeia. Therefore, it is recommended to search these two columns first when choosing keywords in the future. On the other hand, it can also be seen that the Baidu index method is used to predict the change trend of the new coronavirus pneumonia. If the keywords are not selected properly, not only will the accuracy of the prediction be low, but sometimes it may even make it impossible to predict in advance.

In addition, we can see that the CDC diagnosis number and Baidu index data have peak times, so we can compare the correlation between the Baidu index data and the CDC-Diagnosis number from the perspective of the first peak generation time and the time difference, which are shown in Table 2. From the comparative analysis of Figure 1 and Table 2, we can draw the following conclusions. The actual number of new coronavirus pneumonia cases in China reached its highest value on 12 February 2020, which was 15,152, while the Baidu Index data all reach their peak before this date, and the average value of the first peak time difference between the Baidu Index data based on the six keywords and the newly diagnosed CDC is 18 days. This is mainly because during the outbreak of the COVID-19, people like to discuss the it on social media networks. The information released on the new crown epidemic is often of good real-time. The CDC data collection comes from the national infectious disease surveillance system, where the pneumonia often requires a longer diagnosis process from onset to diagnosis, usually 7–14 days.

#### 2.1.2. The Influence of the Selected Index on the Result

**Figure 2:**
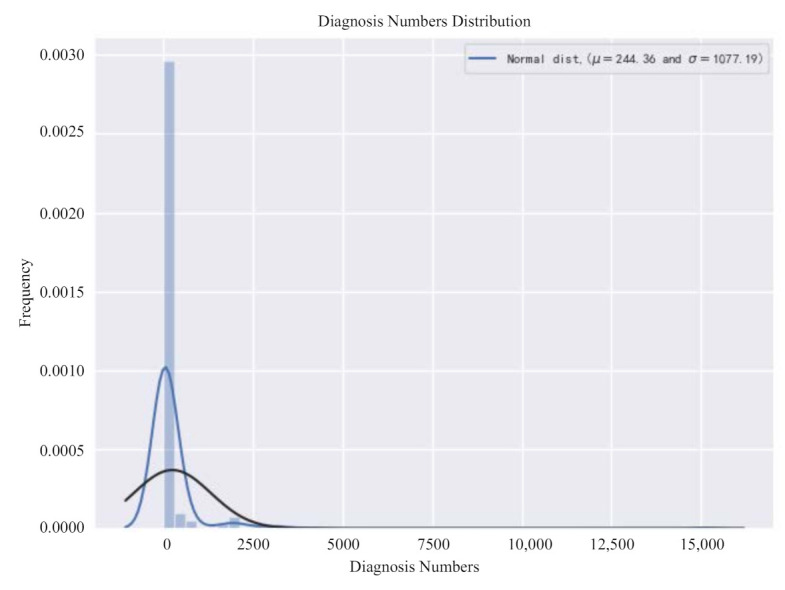
Original diagnosis numbers distribution diagram.

In order to explore the impact of the selected index on the results, we first need to check the distribution of the number of new coronavirus confirmed in the data, as shown in Figure 2. It can be seen from the figure that the overall distribution of the target variable deviates from the normal distribution and needs to be adjusted later. The skewness and kurtosis are calculated again, and the calculation results are 10.72 and 140.84, respectively. It can basically be determined that the skewness of the data in this paper is relatively large and needs to be adjusted.

**Figure 3:**
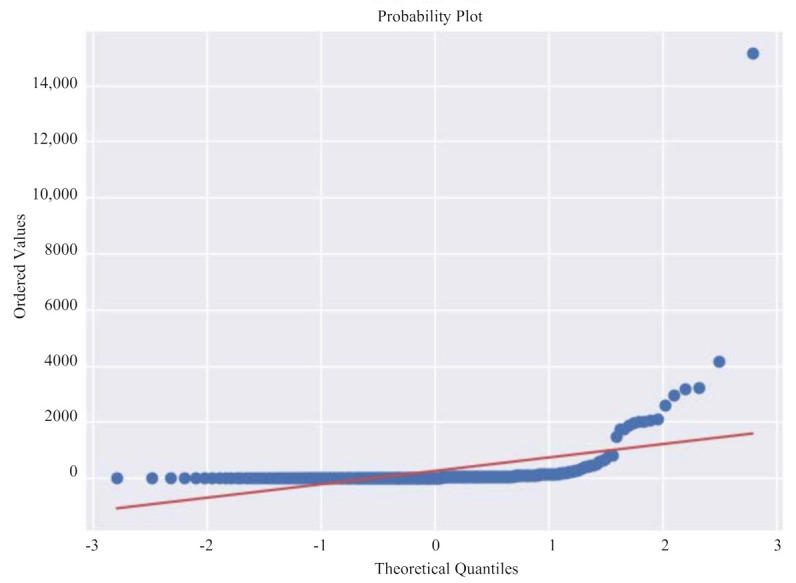
Original diagnosis numbers Q-Q diagram.

Figure 3 shows the Q-Q graph of the COVID-19 data set. Judge whether the data conforms to the normal distribution by comparing whether the quantiles of the data and the normal distribution are equal. The red line represents the normal distribution, and the blue line represents the sample data. The closer the blue and red reference lines are, the more in line with the expected distribution. From the distribution of data, the data presents a normal state. It is further verified that the data distribution has a large skewness, and further data conversion is needed to make it conform to the normal distribution.

**Figure 4:**
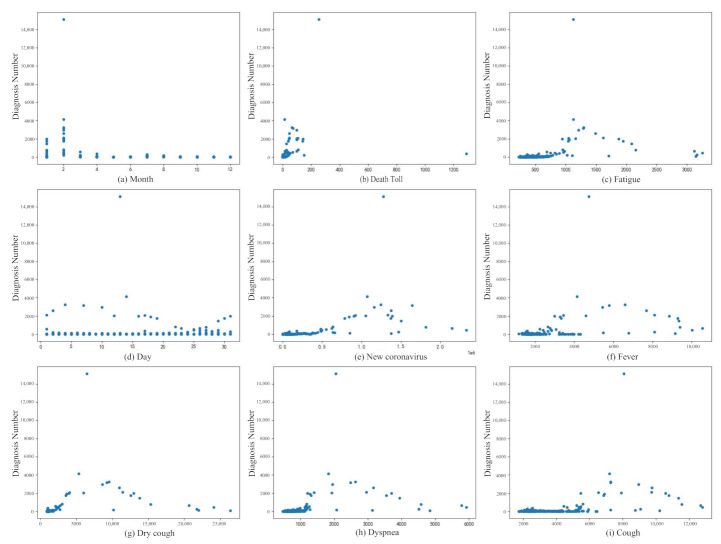
The impact of all attributes on diagnosis numbers. (**a**,**d**) show the trend of newly diagnosis number by month and day, respectively. (**b**) represents the relationship between the diagnosis numbers and the death toll. (**e**) represents the relationship between the diagnosis numbers and new diagnosis released by the CDC. (**c**,**f**,**g**,**h**) and (**i**) respectively represent the relationship between the diagnosis numbers and Baidu Index data based on keyword search, and they correspond to keywords “Fatigue” “Fever” “Dry cough” “Dyspnea” and “Cough” respectively.

**Figure 5:**
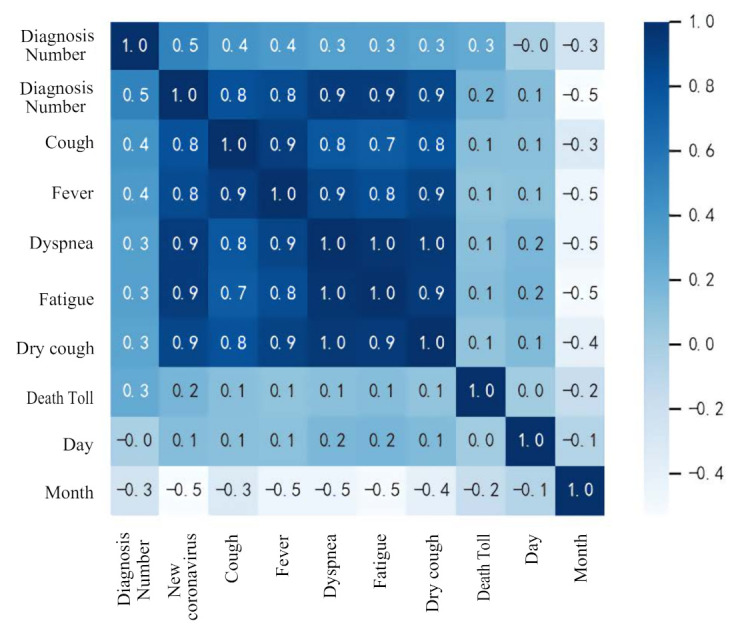
Heat map between variables.

Figure 4 shows the relationship between Diagnosis Numbers and other attributes. It can be seen from the figure that the attributes in the data set are basically positively correlated with the attributes of Diagnosis Numbers. Figure 5 shows the relationship between all attributes, which can be represented by a heat map. The heat map uses different colors to intuitively show the relationship between different attributes, which is a very simple way of data interpretation. The values in the figure are calculated using Pearson’s correlation coefficient. The calculation formula of Pearson’s correlation coefficient is

(1) $$r\left(X,Y\right)=\frac{\mathrm{Cov}(X,Y)}{\sqrt{\mathrm{Var}[X]\mathrm{Var}[Y]}}.$$

It can be seen from the heat map that the attribute of month is negatively correlated with Diagnosis Numbers. It can be seen from the above analysis that the collected data set has a certain influence on Diagnosis Numbers and can be used for the numerical prediction of Diagnosis Numbers.

### 2.2. RF-BOA-LightGBM

As a new cutting-edge technology, predictive models based on machine learning have been widely used in various fields of medicine. For example, Y. D. Zhang et al. proposed a new attention network model, namely ANC (attention network for COVID-19) model, which can diagnose COVID-19 more effectively and accurately [9]. X. Zhang et al. enhanced the deep learning network AlexNet to achieve a more effective classification of new coronary pneumonia [10]. Here, we consider using the RF-BOA-LightGBM (random forest-Bayesian optimization algorithm-light gradient boosting machine) model to predict the development trend of the COVID-19.

#### 2.2.1. Model Structure

**Figure 6:**
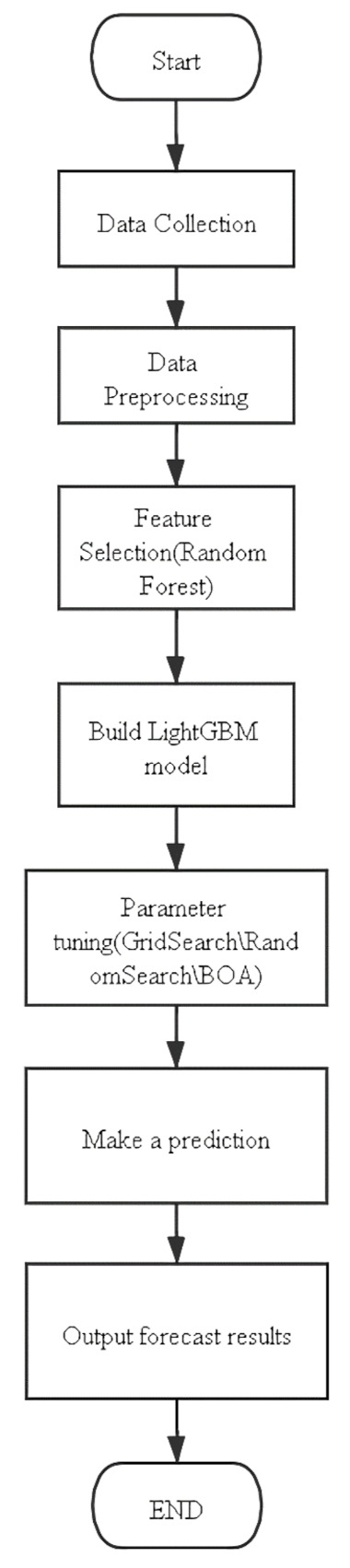
RF-BOA-LightGBM structure. BOA = Bayesian optimization algorithm.

Figure 6 shows the model structure used in this article. After collecting the data, you need to perform a simple processing on the data, so that this model can “learn” the data. Then build the LightGBM model for training, but due to the many parameters of LightGBM, the effect of using the default parameters to train the data set in this article is not necessarily good, so three hyperparameter tuning algorithms are introduced here to adjust the model parameters of LightGBM Perform tuning. After finding a combination of model parameters suitable for the data set in this article, the training prediction is carried out.

#### 2.2.2. Dataset Preprocessing

In order to enable the model to fully learn the data obtained from the Baidu Index COVID-19 vaccine, this article first made great efforts to preprocess the data. It can be seen from the foregoing that the distribution of the data in this paper presents a similar normal distribution. Therefore, this article first performs logarithmic transformation on the data to make the data satisfy the normal distribution. The data conversion formula is

(2) $$y=\log_{c}\left(1+\lambda_{x}\right).$$

Then, deal with the missing data in the data set and delete the samples with missing values (there are not many samples with missing values, which has little effect on the results). Subsequently, the date is divided into three attributes: year, month, and day, and the year attribute is deleted (the year attribute is a fixed value and has little effect on the result), which avoids the problem that the model cannot directly process the date. Finally, the maximum and minimum normalization method is used to integrate the data into (0, 1) range data, which eliminates the influence between samples of different orders of magnitude. The maximum and minimum normalization formula is as follows

(3) $$X_{\mathrm{norm}\;}=\frac{X-X_{\min}}{X_{\max}-X_{\min}}.$$

**Figure 7:**
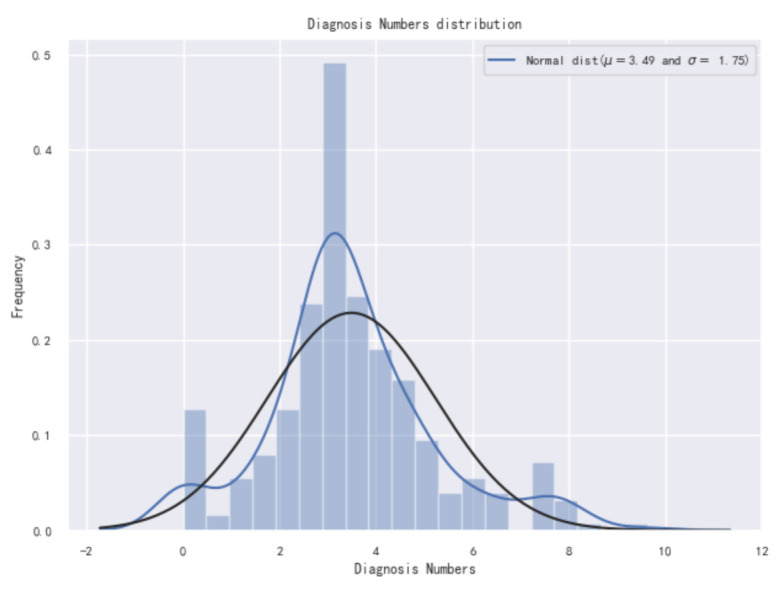
Distribution of diagnosis numbers after data conversion.

**Figure 8:**
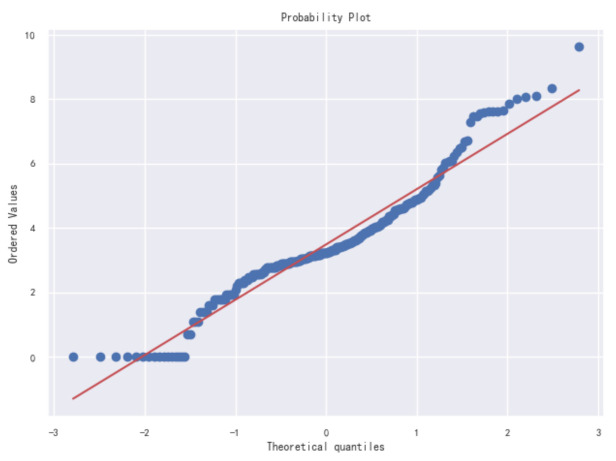
Diagnosis numbers Q-Q diagram after data conversion.

The distribution graph and Q-Q graph of the processed data are shown in Figure 7 and Figure 8 respectively. As can be seen from the figure, the data has basically satisfied the normal distribution.

This data set contains feature data related to the number of new crowns, irrelevant feature data and related but redundant feature data. In the face of complex faults, it is no longer possible to accurately obtain the number of new crowns by relying only on expert experience and simple correlation analysis to perform feature selection work. Important features, so this article uses random forest (RF) out-of-bag estimation to rank the importance of new crown-related features. The random forest is used to select the features of the data set, and the features that have little influence on the prediction results are eliminated.

RF is a combined classifier based on decision trees, which can be used for feature selection [11]. RF uses the Bagging method to randomly and repeatably extract samples from the original sample set for classifier training. About 1/3 of the sample data will not be selected [12]. This data is called Out of Bag (OOB). When calculating the importance of a certain feature, use the OOB data as the base learner after the test set to test the training, and the test error rate is recorded as the out-of-bag error (errOOB). Add noise to the important features to be calculated in the OOB sample, and recalculate errOOB again. The average test error of all base learners is calculated by using the average accuracy decrease rate (MDA) as an indicator for feature importance calculation, namely

(4) $$MDA=\frac{1}{n}\sum_{t=1}^{n}\left(\mathrm{err}OOB_{t}-{\mathrm{errOOB}}_{t}^{\prime }\right),$$

where *n* is the number of base learners, errOOB is the out-of-bag error after adding noise.

The more the MDA index decreases, the more the corresponding feature has a greater impact on the prediction result, and the higher its importance. This feature importance calculation method is called random forest out-of-bag estimation. According to this method, the importance of fault-related features is ranked and feature selection is performed.

#### 2.2.3. Tuning Algorithm

For the LightGBM model, there are many internal hyperparameters that affect the prediction results. However, if the value of the hyperparameter used is the default value, this hyperparameter combination may not be the optimal hyperparameter combination for the new coronavirus number prediction data set [13]. Therefore, this paper introduces three tuning algorithms, namely grid search, random search, and Bayesian optimization, to optimize some important hyperparameters of LightGBM [14]. Before adjusting the parameters of LightGBM, the optimization range of hyperparameters is generally set first. These three algorithms are briefly described below.

Grid search divides the search range into grid shapes, and adjusts the parameters according to the set step to train the model until all possible combination parameters are verified, and finally the parameter combination that gives the best result is output [15]. Because the different prediction results of the data in each group of hyperparameter combinations are also different, when the hyperparameter combination is relatively large and the search range is relatively large, the optimization speed of the grid search is very slow.

Random search is similar to grid search, but it does not verify all possible parameter combinations like grid search, but randomly combines the random value of each parameter, so the speed of random search is faster than that of Grid search [16]. However, random search may also miss the parameter combination that maximizes the prediction result.

Bayesian optimization algorithm(BOA) can quickly find the optimal parameters for the problem to be solved based on historical experience [17]. The main problem scenarios for Bayesian optimization are

(5) $$X^{*}=\mathrm{argmax}f\left(x\right)\left(x\in S\right),$$

where *x* is the parameter to be optimized, *S* is the candidate set of *x* variable, that is, the set of possible values of parameter *x*. The target selects an *x* from the set *S* such that the value of f(x) is the largest or smallest. Here, the specific formula of f(x) may not be known, that is, the black box function. But you can choose an *x*, and get the value of f(x) through experiment or observation [18].

BOA has two core processes, a priori function (PF) and acquisition function (AC). The acquisition function is also called the efficiency function. Under the framework of Bayesian decision theory, many collection functions can be interpreted as evaluating the expected loss associated with *f* at point *x*, and then usually selecting the point with the lowest expected loss [19]. PF mainly uses Gaussian process regression, AC mainly uses these methods including EI (expected improvement), PI (probability of improvement) and UCB (upper confidence bound), and this article uses the EI function. The EI function can find out the global optimum without falling into the local optimum. The collection function is as follows

(6) $$u\left(x\right)=\max\left(0,f^{\prime }-f\left(x\right)\right),$$

where *f* is the collection function, and f(x) is the optimized performance indicator.

The final collection function for variable *x* is

(7) $$\begin{array}{c} a_{\mathrm{EI}}\left(x\right)=E\left[u\left(x\right)|x,D\right]=\int_{-\infty }^{f^{\prime }}\left(f^{\prime }-f\right)N\left(f;u\left(x\right),K\left(x,x\right)\right)df\\ \left.=\left(f^{\prime }-u\left(x\right)\right)\Phi \left(f^{l};u\left(x\right),K\left(x,x\right)\right)+K\left(x,x\right)\right)N\left(f^{\prime };u\left(x\right),K\left(x,x\right)\right). \end{array}$$

The calculation shows that the point corresponding to the maximum value of $a_{\mathrm{EI}}$ is the best point. There are two components in Formula (7). To maximize the value of it, you need to optimize the left and right parts at the same time, that is, the left side needs to reduce the μ(x) as much as possible, and the right side needs to increase the variance (or covariance) *K*(*x*, *x*) as small as possible. It is a typical theory on issues such as exploration and exploitation.

Upper confidence bound (UCB) can be simply understood as the upper confidence boundary. It is usually described by maximizing *f* instead of minimizing *f*. But in the case of minimization, the collection function will take the following form

(8) $$a_{UCB}\left(x\right)=u\left(x\right)-\beta \sigma \left(x\right),$$

where β>0 is a strategy parameter, and $\sigma \left(x\right)=\sqrt{K(x,x)}$ is the boundary standard deviation of f(x). Similarly, UCB also includes exploitation (u(x)) and exploration ((x) modes. It can converge to the global optimal value under certain conditions.

Table 3 shows the hyperparameter combinations selected in this article and the corresponding descriptions.

#### 2.2.4. LightGBM

LightGBM is an open source decision tree-based gradient boosting framework proposed by Microsoft. As an improved version of Gradient Boosting, it has the characteristics of high accuracy, high training efficiency, support for parallelism and GPU, small memory required, and ability to handle large-scale data [20].

According to the different generation methods of the base learner, integrated learning can be divided into parallel learning and serial learning. As the most typical representative of serial learning, Boosting algorithm can be divided into Adaboost and Gradient Boosting. The main difference between them is that the former improves the model by increasing the weight of misclassified data points, while the latter improves the model by calculating negative gradients. The core idea of Gradient Boosting is to use the negative gradient of the loss function to approximate the value of the current model $f\left(x\right)=f_{j-1}\left(x\right)$ to replace the residual. Suppose the training sample is *i* (i=1,2,…,n), the number of iterations is *j* (j=1,2,…,m), and the loss function is L(yi,f(xi)), then the negative gradient $r_{ij}$ can be expressed as

(9) $$r_{ij}=-{\left[\frac{\partial L\left(y_{i},f\left(x_{i}\right)\right)}{\partial f\left(x_{i}\right)}\right]}_{f\left(x\right)=f_{j-1}\left(x\right)}.$$

Use the base learner $h_{j}\left(x\right)$ to fit the negative gradient *r* of the loss function, and find the best fit value $r_{j}$ that minimizes the loss function

(10) $$r_{j}=\arg\min L\left(y_{i},f_{j-1}\left(x_{i}\right)+rh_{j}\left(x_{i}\right)\right).$$

Model update:

(11) $$|f_{j}\left(x\right)=f_{j-1}\left(x\right)+r_{j}h_{j}\left(x\right).$$

Gradient Boosting generates a base learner in each round of iteration. Through multiple rounds of iteration, the final strong learner F(x) is the base learner generated in each round and obtained by linear addition:

(12) $$F\left(x\right)=f_{m}\left(x\right)$$

As an improved lightweight Gradient Boosting algorithm, the core ideas of LightGBM are: histogram algorithm, leaf growth strategy with depth limitation, direct support for category features, histogram feature optimization, multithreading optimization, and cache hit rate optimization. The first two features effectively control the complexity of the model and realize the lightweight of the algorithm, so this article is particularly concerned.

The histogram algorithm discretizes continuous floating-point features into *L* integers to construct a histogram with a width of *L*. When traversing the data, use the discretized value as an index to accumulate statistics in the histogram. After traversing the data once, the histogram accumulates the necessary statistics, and then find the optimal split point from the discrete values of the histogram .

The traditional leaf growth strategy can split the leaves of the same layer at the same time. In fact, the splitting gain of many leaves is low and there is no need to split, which brings a lot of unnecessary expenses. For this, LightGBM uses a more efficient leaf growth strategy: each time it searches for the leaf with the largest split gain from all the current leaves to split, and sets a maximum depth limit. While ensuring high efficiency, it also prevents the model from overfitting.

## 3. Results and Discussion

### 3.1. Performance Predictor

All models are cross-validated and the coefficient of determination (R2), mean absolute error (MAE), relative absolute error (RAE), relative square root error (RRSE), root mean square error (RMSE) are calculated, as shown below

(13) $$R2\left(y,\hat{y}\right)=\sqrt{1-\frac{\sum_{i=1}^{n}{\left(y_{i}-{\hat{y}}_{i}\right)}^{2}}{\sum_{i=1}^{n}{\left(y_{i}-\bar{y}\right)}^{2}}},$$

(14) $$\mathrm{RMSE}\left(y,\hat{y}\right)=\sqrt{\frac{1}{n}\sum_{i=1}^{n}y_{i}-{\hat{y}}_{i}^{2}},$$

(15) $$\mathrm{MAE}\left(y,\hat{y}\right)=\frac{1}{n}\sum_{i=1}^{n}\left|y_{i}-{\hat{y}}_{i}\right|,$$

(16) $$\mathrm{RAE}\left(y,\hat{y}\right)=\sqrt{\frac{\sum_{i=1}^{n}\left|y_{i}-{\hat{y}}_{i}\right|}{\sum_{i=1}^{n}\left|y_{i}-\bar{y}\right|}},$$

(17) $$\mathrm{RRSE}\left(y,\hat{y}\right)=\sqrt{\frac{\sum_{i=1}^{n}{\left(y_{i}-{\hat{y}}_{i}\right)}^{2}}{\sum_{i=1}^{n}{\left(y_{i}-\bar{y}\right)}^{2}}},$$

where *y* represents the true value, $\hat{y}$ represents the predicted value, $\bar{y}$ represents the average value of the true value and *n* is the number of test sets.

### 3.2. Experiment Results

**Figure 9:**
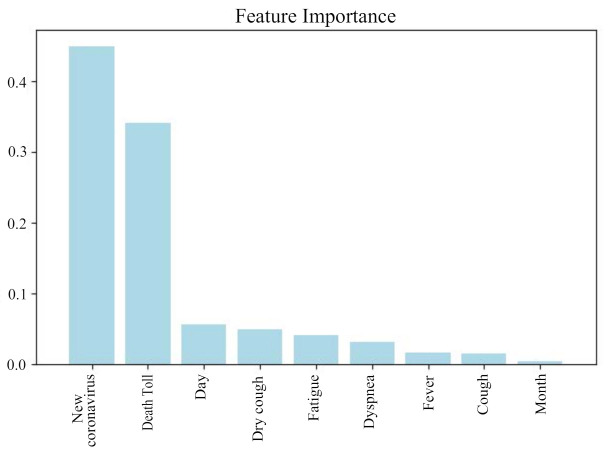
Feature selection results.

Figure 9 shows the result of feature selection using random deep forest, and the features are output in descending order of importance. It can be seen from the figure that Death Toll has the greatest impact on Diagnosis Numbers, while the attribute of Month has the least impact. Finally, we selected the 7 most influential attributes for the prediction of Diagnosis Numbers.

According to the optimal parameter set of the model, the Diagnosis Numbers prediction model of COVID-19 is constructed. In this paper, LightGBM, GridSearch-LightGBM, RandomSearch-LightGBM, and BOA-LightGBM models are used for Diagnosis Numbers prediction. Table 4 shows the specific values of the optimal parameter combinations found by the three tuning algorithms.

Table 5 shows the evaluation indicators of the prediction results of the four models. The prediction results of the model are evaluated by R2, RMSE, MAE, RAE, RRSE evaluation indicators. It can be seen from the values of the five evaluation indicators that the results of BOA-LightGBM are better than the former. RandomSearch-LightGBM and GridSearch-LightGBM have their own advantages and disadvantages. It can also be seen that the default hyperparameters of LightGBM are not suitable for the prediction of Diagnosis Numbers of COVID-19 in this article. From the approximate prediction effect, BOA-LightGBM can better analyze the relationship between historical data and can effectively predict the value of Diagnosis Numbers of COVID-19, which proves the superiority of the model.

**Figure 10:**
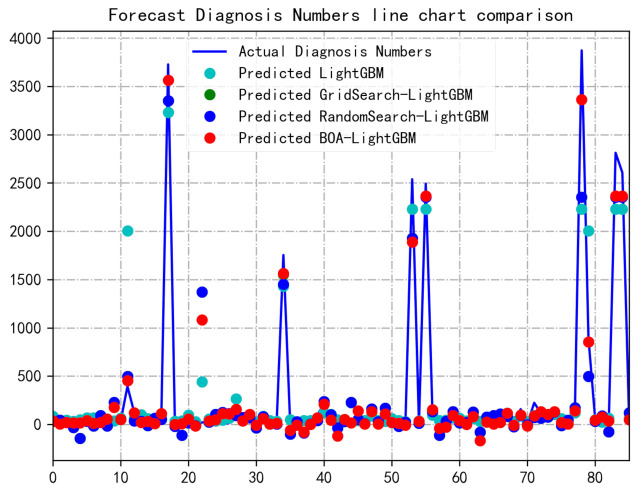
Comparison of predicted and true values of the four models. BOA, Bayesian optimization algorithm; GBM, gradient boosting machine.

Figure 10 is a line chart of the four algorithms to predict Diagnosis Numbers, and only part of the data is taken on the abscissa. The prediction effect of the model can be seen more intuitively from the line graph. It can be seen from the figure that in most cases, the BOA-LightGBM model can better fit the fluctuation trend of Diagnosis Numbers at some points, and the predicted value is very close to the actual value. In the figure, the points predicted by GridSearch-LightGBM are basically covered, so they are not shown in the figure, which just shows that the prediction results are not very prominent. Sometimes the prediction value of LightGBM is better than other models, but most of them are inferior to other models. So comprehensively, the BOA-LightGBM model is more in line with the changing trend of real values.

## 4. Conclusions

This study uses the Internet big data tool-Baidu Index to predict the development trend of the new coronavirus pneumonia epidemic to obtain data. By selecting appropriate keywords, data on COVID-19 cases in China from 1 January 2020 to 1 April 2020 are collected. After preprocessing the data set, the random forest feature selection method is used to obtain the optimal sub-data set. After comparing and analyzing the optimization results of the seven hyperparameters of the LightGBM model with the three optimization algorithms of grid search, random search, and Bayesian optimization. It is concluded that applying the data set obtained from the Baidu Index to the Bayesian-optimized LightGBM model can better predict the increase in the number of new coronary pneumonias, and it is a good aid to predict the new number of new coronary pneumonia in the future medical structure effect.

## Figures and Tables

**Table 1:** Partial data from CDC and Baidu Index search.

		Source	CDC- Diagnosis	Baidu- New Coronavirus	Baidu- Fever	Baidu- Dry Cough	Baidu- Fatigue	Baidu- Dyspnea	Baidu- Cough	CDC- Death Toll
	Data									
Date										
1 January 2020			0	0	4001	1100	256	481	5885	0
2 January 2020			0	0	4323	1206	278	602	6448	0
3 January 2020			1	0	4212	1173	262	654	6392	0
4 January 2020			0	0	4309	1109	270	621	6570	0
5 January 2020			5	0	4327	1118	271	591	6564	0
6 January 2020			0	0	4324	1226	310	693	6404	0
7 January 2020			0	0	3920	1175	288	633	5875	0
8 January 2020			0	0	3803	1124	272	622	5354	0
9 January 2020			0	8812	3693	1131	270	579	5182	0
10 January 2020			0	2032	3700	1095	263	535	5022	0
11 January 2020			0	2879	3478	1083	237	498	5033	1
12 January 2020			0	1445	3364	1067	252	474	5011	1
13 January 2020			0	1515	3573	1118	278	494	4418	1
14 January 2020			0	4846	3479	1133	266	528	4359	1
15 January 2020			0	4191	3241	1097	245	512	4355	2

Note: CDC = Centers of Disease Control.

**Table 2:** Comparison of peak time between Baidu Index data based on different keywords and CDC new diagnostic data.

Category	First Peak Time	Time Difference (Days)
CDC-Diagnostic	12 February 2020	-
Baidu-New coronavirus	25 January 2020	+18
Baidu-Fever	26 January 2020	+17
Baidu-Dry cough	23 January 2020	+20
Baidu-Fatigue	25 January 2020	+18
Baidu-Dyspnea	25 January 2020	+18
Baidu-Cough	25 January 2020	+18
Arithmetic mean	-	+18

Note: + indicates the number of days in advance, - indicates the number of days later. CDC = Centers of Disease Control.

**Table 3:** The LightGBM hyperparameters selected in this article and their functions.

Parameter	Style	Search Scope	Effect
learn_rate	float	(0.001, 0.3)	improve accuracy
max_depth	int	(3, 10)	prevent overfitting
num_leaves	int	(3, 1024)	improve accuracy
min_data_in_leaf	int	(0, 80)	prevent overfitting
feature_fraction	float	(0.2, 0.9)	accelerate
bagging_fraction	float	(0.2, 0.9)	accelerate
lambda_l1	float	(0, 10)	prevent overfitting

**Table 4:** Specific parameter values found by three tuning algorithms.

Parameter	GridSearch	RandomSearch	BOA
learn_rate	0.632	0.828	0.355
max_depth	7	8	5
num_leaves	225	237	249
min_data_in_leaf	33	27	30
feature_fraction	0.7	0.7	0.8
bagging_fraction	0.7	0.7	0.8
lambda_l1	2.34	3.45	1.80

Note: BOA = Bayesian optimization algorithm.

**Table 5:** Model evaluation index.

Models	R2	RMSE	MAE	RAE	RRSE
LightGBM	0.820	354.945	138.939	0.535	0.424
GridSearch-LightGBM	0.865	311.918	145.266	0.548	0.368
RandomSearch-LightGBM	0.861	316.217	137.621	0.533	0.373
BOA-LightGBM	0.879	295.686	124.911	0.508	0.348

Note: GBM, gradient boosting machine; BOA, Bayesian optimization algorithm; R2, coefficient of determination; RMSE, root mean square error; MAE, mean absolute error; RAE, relative absolute error; RRSE, relative square root error.

## Data Availability

Data are available at School of Life Sciences, Central South University, China.

## Appendix A. Datasets from CDC and Baidu Index Search

**Table A1:** Datasets from CDC and Baidu Index search.

		Source	CDC- Diagnosis	Baidu- New Coronavirus	Baidu- Fever	Baidu- Dry Cough	Baidu- Fatigue	Baidu- Dyspnea	Baidu- Cough	CDC- Death Toll
	Data									
Date										
1 January 2020			0	0	4001	1100	256	481	5885	0
2 January 2020			0	0	4323	1206	278	602	6448	0
3 January 2020			1	0	4212	1173	262	654	6392	0
4 January 2020			0	0	4309	1109	270	621	6570	0
5 January 2020			5	0	4327	1118	271	591	6564	0
6 January 2020			0	0	4324	1226	310	693	6404	0
7 January 2020			0	0	3920	1175	288	633	5875	0
8 January 2020			0	0	3803	1124	272	622	5354	0
9 January 2020			0	8812	3693	1131	270	579	5182	0
10 January 2020			0	2032	3700	1095	263	535	5022	0
11 January 2020			0	2879	3478	1083	237	498	5033	1
12 January 2020			0	1445	3364	1067	252	474	5011	1
13 January 2020			0	1515	3573	1118	278	494	4418	1
14 January 2020			0	4846	3479	1133	266	528	4359	1
15 January 2020			0	4191	3241	1097	245	512	4355	2
16 January 2020			0	5174	3230	1100	267	546	4220	2
17 January 2020			4	7713	3247	1114	254	521	4008	2
18 January 2020			17	7754	3271	1060	228	492	4218	2
19 January 2020			36	29,003	3418	1182	253	548	4323	2
20 January 2020			151	266,892	4064	3684	609	1090	5324	2
21 January 2020			77	659,926	5474	10,162	1106	2073	7260	2
22 January 2020			149	852,363	6782	21,967	1711	3125	8751	3
23 January 2020			131	1,374,253	9151	26,393	3141	4840	10,229	11
24 January 2020			259	1,469,947	8108	21,718	3162	4511	9059	41
25 January 2020			688	2,330,851	10029	24,100	3253	5922	12798	56
26 January 2020			769	2,150,021	10552	20,635	3117	5779	12677	80
27 January 2020			1771	1,816,430	9406	15,323	2152	4572	11,547	106
28 January 2020			1459	2,227,942	9091	15,115	2296	4087	11,185	132
29 January 2020			1737	1,503,255	9350	13,783	2088	3940	11,351	170
30 January 2020			1982	1,372,206	9287	12,574	1943	3541	10,786	213
31 January 2020			2102	1,390,560	8855	12,974	1876	3702	10,584	259
1 February 2020			2590	1,334,127	8108	11,425	1620	2952	9741	304
2 February 2020			2829	1,374,154	7682	10,981	1491	3162	9750	361
3 February 2020			3235	1,277,132	7258	10,683	1365	2949	8517	425
4 February 2020			3887	1,244,048	6602	9504	1293	2626	7258	490
5 February 2020			3694	1,209,808	6213	8763	1349	2380	7434	563
6 February 2020			3143	1,943,197	5736	8305	1295	2179	8043	636
7 February 2020			3399	1,643,941	5789	9236	1292	2488	7261	722
8 February 2020			2656	1,185,978	5126	7287	1183	2131	6718	811
9 February 2020			3062	1,142,892	5220	8719	1187	2004	8173	908
10 February 2020			2478	1,158,302	5450	8585	1212	1946	8948	1016
11 February 2020			2015	1,061,433	4814	7421	1239	1901	8641	1113
12 February 2020			15,152	1,050,392	4590	5971	1163	1922	7908	1367
13 February 2020			5090	1,277,024	4745	6436	1125	2049	8076	1380
14 February 2020			2641	1,069,203	4140	5339	1126	1830	7197	1523
15 February 2020			2009	948,165	3295	4537	1018	1456	6452	1596
16 February 2020			2048	904,431	2994	3953	942	1205	5461	1770
17 February 2020			1886	920,373	3454	4025	1046	1406	6542	1868
18 February 2020			1749	840,490	3274	3652	1056	1278	6889	2004
19 February 2020			394	784,784	3327	3530	1038	1315	6848	2118
20 February 2020			889	800,960	3035	3071	1012	1345	6552	2236
21 February 2020			397	776,563	3003	3244	935	1269	6467	2345
22 February 2020			648	636,594	2663	3003	949	1179	5606	2442
23 February 2020			409	622,095	2777	2771	978	1172	5218	2592
24 February 2020			508	634,391	3234	2695	1025	1286	5940	2663
25 February 2020			406	550,484	3066	2550	964	1260	5462	2715
26 February 2020			433	482,726	2850	2468	896	1202	5451	2744
27 February 2020			327	478,822	2835	2403	819	1165	5354	2788
28 February 2020			427	486,394	2660	2285	845	1195	5425	2835
29 February 2020			573	496,289	2420	2213	750	1133	4655	2870
1 March 2020			202	482,280	2244	2070	686	1151	4458	2912
2 March 2020			125	441,914	2468	2123	785	1176	5326	2943
3 March 2020			119	393,118	2223	1955	755	1143	4741	2981
4 March 2020			139	441,921	2264	1970	765	1163	4967	3012
5 March 2020			143	414,142	2122	1789	680	1140	5157	3042
6 March 2020			99	376,106	2111	1658	694	1112	5186	3070
7 March 2020			44	369,780	1877	1539	723	1072	4196	3097
8 March 2020			40	368,916	1759	1480	646	1052	3993	3119
9 March 2020			19	359,426	2017	1414	687	1133	5547	3136
10 March 2020			24	335,711	1792	1288	635	1085	4164	3158
11 March 2020			15	337,491	1911	1413	633	1049	4331	3169
12 March 2020			8	353,167	1891	1575	686	1088	3967	3176
13 March 2020			11	353,857	1906	1756	641	1119	3269	3189
14 March 2020			20	332,215	1745	1358	601	1042	2788	3199
15 March 2020			16	364,033	1721	1486	657	1037	2732	3213
16 March 2020			21	324,566	1985	1555	759	1087	3845	3226
17 March 2020			13	300,185	1885	1546	673	1068	3022	3237
18 March 2020			34	295,536	1920	1491	696	1052	3198	3245
19 March 2020			39	282,990	1724	1355	663	1057	2742	3248
20 March 2020			41	300,183	1779	1227	621	1036	2705	3255
21 March 2020			46	299,291	1734	1308	641	1006	2577	3261
22 March 2020			39	285,191	1736	1102	672	1027	2829	3270
23 March 2020			78	280,841	1855	1391	704	1102	3018	3277
24 March 2020			47	278,221	1830	1457	704	1052	3215	3281
25 March 2020			67	259,091	1810	1308	656	1045	3446	3287
26 March 2020			55	261,957	1839	1094	655	1091	3114	3292
27 March 2020			54	279,082	1645	1129	592	1061	2780	3295
28 March 2020			45	264,664	1525	1065	476	998	2480	3300
29 March 2020			31	265,761	1562	1096	490	961	2364	3304
30 March 2020			48	264,442	1725	1094	601	1031	3021	3305
31 March 2020			36	239,272	1772	1071	535	1007	2676	3312
1 April 2020			35	243,582	1569	1080	565	1013	2676	3318

Note: CDC = Centers of Disease Control.
